# Combined Crack Initiation and Crack Growth Model for Multi-Layer Polymer Materials

**DOI:** 10.3390/ma15093273

**Published:** 2022-05-03

**Authors:** Martin Pletz, Florian Josef Arbeiter

**Affiliations:** 1 Designing Plastics and Composite Materials, Montanuniversitaet Leoben, 8700 Leoben, Austria; 2 Materials Science and Testing of Polymers, Montanuniversitaet Leoben, 8700 Leoben, Austria

**Keywords:** multi-layer materials, elastic-plastic fracture mechanics, crack initiation, polymer materials, finite fracture mechanics

## Abstract

The current publication deals with the fracture toughness of polymeric multi-layer materials. In detail, the crack initiation and growth, crack arrest, and crack re-initiation of a multi-layer material are examined. The aim is to develop a numerical model for crack initiation and incremental crack growth of a three-layer single edge notched bending specimen that features one brittle layer in a plastically deforming matrix. Crack initiation and crack propagation are modeled using the finite fracture mechanics concept and the energy concept, respectively. No delamination is accounted for and the crack grows in one plane. The experimental observation of a crack initiating in the brittle layer (at 61.4 ± 2.2 N) while the initial crack is blunting can be reproduced well with the numerical model (at 63.6 N) with a difference of <3.6%. The model is ready to be used for different layups to predict toughening mechanisms and damage tolerances in multi-layer materials.

## 1. Introduction

Multi-layer materials, consisting of different materials and complex architectures, possess many beneficial properties. In nature, many examples of optimized materials can be found. Especially interesting for mechanical engineers is the potential to vastly increase the fracture toughness of materials by applying principles found in nature to engineering materials [1,2,3]. Promising results on both metals [4] and ceramics [5,6,7] have sparked the interest of polymer engineers. Conventional methods such as multi-layer extrusion [8,9,10,11] and emerging technologies, such as additive manufacturing [12,13,14,15,16], are well equipped for the generation of interesting multi-layer materials.

In the field of polymers, many applications of multi-layered structures, such as printed circuit boards, classical carbon fiber reinforced composites, pipes, and containers for media transport, are already in use. To date, however, multi-layer build-ups are mainly used for the functional properties of the added materials and not to optimize mechanical properties, such as fracture toughness. A classical example of the former group is the enhancement of barrier properties, as seen in food-related packaging and fuel storage [17]. Some applications, such as multi-layer pipes in sewer applications, already utilize different materials for their mechanical properties or necessary chemical resistance. However, the exact architecture of the layers is mainly based on the experience of engineers, and extensive and expensive trial and error approaches. In order to optimize the possibilities of multi-layered structures with regard to their mechanical properties, easier and more precise methods are required. Adequate methods have to account for local damage mechanisms such as crack propagation, crack arrest at layers, and subsequent re-initiation.

One possibility to optimize layered materials for structural applications is the use of fracture mechanical approaches. For example, mathematical equations describe the behavior of cracks that are in the vicinity of an interface between materials of different mechanical properties. An example of this approach is the description by He and Hutchinson of crack deflection at an interface between dissimilar elastic materials [18,19], which is a favored model for the description of layered ceramics [20,21].

In contrast to metals and ceramics, polymers often show a very high amount of plastic deformation before fracture under monotonic loading. Therefore, classical methods that are applied to metals and ceramics for the description of the fracture toughness, such as the stress intensity factor, cannot be applied directly.

For inhomogeneous materials there is also the issue of crack deflection and/or crack arrest due to inhomogeneous stress fields or toughness, phase interface debonding, and initiation of cracks from microstructural features. For layered polymers with good adhesion and a straight crack perpendicular to the layer orientation, it can be assumed that only straight crack growth is relevant. Nevertheless, this crack growth can be severely influenced by the stress fields and plastic deformations of the layers.

Currently, different groups are working on approaches to solve the issue of changing crack-driving forces in layered materials. One possibility is the use of configurational-force-based concepts [22], which have shown promising results for several material classes, including polymers [9]. However, they are limited to applications where plastic strains in the vicinity of the crack tip can be separated from plastic strain fields at the scale of the specimen [23]. For very ductile polymers and high levels of plastic deformation, this condition may not be fulfilled. For example, in [9], separate cohesive zone elements had to be introduced to model the initiation of cracks in the brittle layer and further propagation of the crack.

Thus, the current work aims to provide an alternative approach for the description of the fracture behavior of multilayered polymeric materials. The chosen method uses the finite fracture mechanics concept [24] for predicting crack initiation, and the energy approach to assess crack growth (the energy release rate of the crack is compared to the critical energy release rate of the material). The finite fracture mechanics concept uses a combined energy-stress criterium to predict the crack initiation, as proposed by Leguillon et al. [25]. The energy approach for crack growth is used to account for high plastic strains and large regions of plastic deformations. In contrast, the configurational force-based concept with incremental plasticity is based on the separation of the crack tip-associated plastic region and the far-field plastic region [9,26].

In order to evaluate the applicability of the proposed modeling method, bending tests on layered plates were performed. In detail, two vastly different polymer materials, one very ductile and compliant, the other very brittle and rather stiff, were used to constitute the multi-material layers. In this way, it was possible to experimentally observe crack initiation, growth, and crack-stop, in addition to the re-initiation of a crack. The simulation method was evaluated by comparing the experimental results to the simulation results, both qualitatively and quantitatively. To keep the model as simple as possible, von Mises’ yield criterion and time-dependent effects were omitted. Considering the simplicity of the proposed method, the crack initiation and growth, crack arrest, and crack re-initiation can be captured accurately.

## 2. Materials and Methods

The subsequent section provides information regarding the used materials, the experimental setup, and numerical models. In addition, possibilities and limitations of the chosen modeling approach are listed.

### 2.1. Materials and Specimens

**Figure 1:**
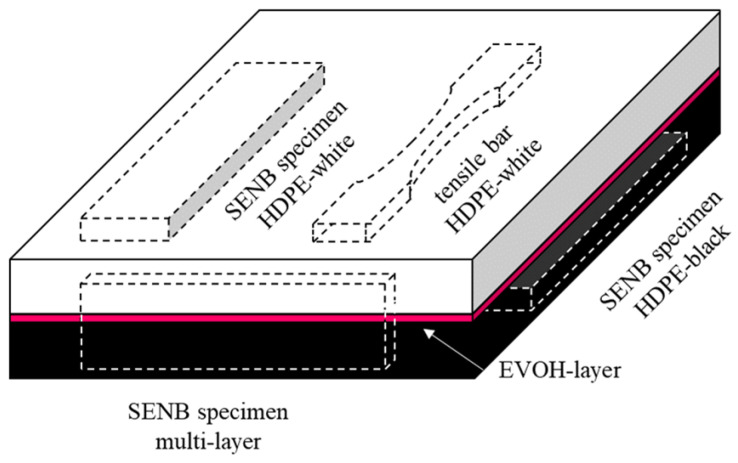
Schematic drawing of the used multi-layer plates and corresponding machined testing specimens. The plate consists of a black HDPE layer, an EVOH layer, and a white HPDE layer at the bottom, the middle, and the top, respectively.

To gauge the applicability of the chosen approach with regard to real-life components, a pre-existing multi-layer component was chosen for the current study. A commercially available co-extruded multi-layer sheet (500 × 500 × 9 mm^3^), consisting of two outer layers of high-density polyethylene (HDPE) and a thin barrier layer of ethylene-vinyl alcohol (EVOH), was used. These types of sheets are commonly used in automotive (e.g., fuel tanks) and other applications where a diffusion barrier is needed to avoid migration of gasoline or other carbon-based liquids. From these plates, specimens were milled for all subsequent tests, as shown in Figure 1. Low milling speeds (esp. rotation) and air-cooling were used to minimize heating of the specimens during milling. No coolant was used to avoid any possible absorption of media into the polymer. Both HDPE-layer (HDPE-black and HDPE-white) tensile test specimens and single-edge notched bending (SENB) specimens were produced. Multi-layer SENB specimens were produced from the front side. Prior to testing, specimens were stored in a standardized climate (23 °C, 50% r.h.) for at least 48 h. No further treatment was performed.

#### 2.1.1. Tensile Testing—Generation of Input Data

The necessary material data for the simulation were acquired by performing tensile tests. Five tensile specimens were cut from both HDPE-black and HDPE-white as shown in Figure 1. The specimens were machined from 50 × 9 × 3.5 mm^3^ bars into dog-bone-shaped specimens with a residual cross-section of 5 × 3.5 mm^2^ in the middle part. Shoulder radii were machined with a radius of 25 mm. The parallel length in the middle was 20 mm. Due to the geometric restrictions of the available multi-layer sheets and machining restrictions, specimens were not prepared according to a specific standard. All tensile tests were performed on a Zwick Z250 (Zwick/Roell, Ulm, Germany), equipped with a 10 kN load cell. To minimize influences due to non-standardized geometry, local strain measurement via digital image correlation (DIC) was used. The specimens were filmed with two cameras from two different angles (front and side). The deformed cross-section of the specimens during testing was calculated from these measurements. Measured strains, force, and deformed cross-section were used to determine true stress vs. true strain curves for the material model of the HDPE material. Strains were evaluated via the Mercury RT system (Sobriety, Brno, Czech Republic). The speckle pattern for DIC was applied via paint-brushing of graphite for white and a combination of graphite and white water-based color (Edding, Ahrensburg, Germany) for the black specimens. The combination of graphite and white color is necessary for black specimens due to the excessive stress whitening at high strains, which would lead to complete loss of the pattern.

#### 2.1.2. Testing of Fracture Mechanical Properties

**Figure 2:**
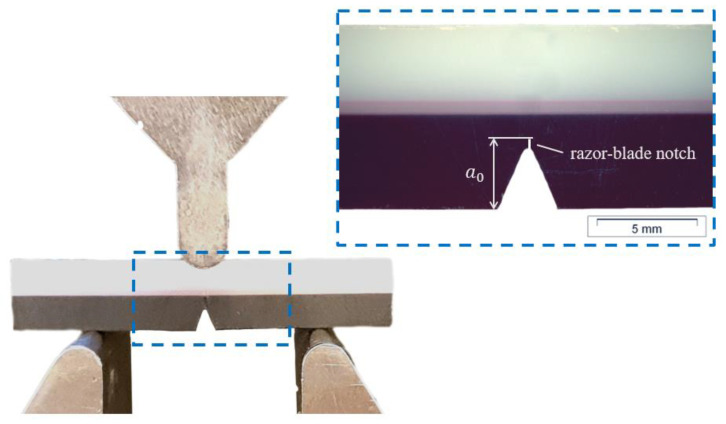
Photograph of a multi-layer SENB specimen with two outer HDPE layers in white and black, and the middle barrier layer consisting of EVOH in pink (before side-grooving). The used three-point bending setup with a support length of 36 mm is also shown.

Fracture mechanical testing of SENB specimens was performed on a MTS831.50 (MTS, Eden Prairie, MN, USA), equipped with a 1 kN load cell. All specimens were notched via broaching, which has shown promising results for HDPE [27]. This type of notching usually yields a notch tip radius of around 1 µm in HDPE. The orientation and position of all SENB specimens in the original multi-layer plate are shown in Figure 1. The geometry of the specimens for homogeneous HDPE was chosen according to the ESIS-TC4 protocol by Hale and Ramsteiner [28] with a width (*W*) of the plate thickness (9 mm), a support span (S) of 4 × *W*, and a specimen thickness (*B*) of *W*/2. The initial crack length (*a*_0_) was chosen as 60% of *W*. In order to promote plane strain conditions, side grooves with a depth of 0.1 × *B* were implemented on both sides. For the multi-layer specimens, the initial crack length was reduced to approximately *a*_0_ = 0.45 × *W* − 0.48 × *W*, in order to enable the crack to propagate towards and subsequently through the interlayer. The testing velocity was chosen as 2 mm/min. A representative multi-layer SENB specimen is shown in Figure 2. Two times six specimens were tested for the single material setup, and six were tested for the multilayer setup.

#### 2.1.3. Fractographical Analysis

To subsequently compare the overall fracture behavior of both experiment and simulation, fractography was performed. Therefore, highly deformed but unbroken specimens were cryo-fractured in liquid nitrogen, similar to single material SENB specimens according to [28], and examined via scanning electron microscopy (SEM). The pictures were taken using a Vega-Tescan II (Tescan, Brno, Czech Republic) with 5 kV, after sputtering the specimens with a gold coating.

### 2.2. Numerical Models

A finite element (FE) model of the tensile test was used to evaluate the constitutive law of the HDPE material that is obtained from the tensile test. The model of the SENB test was developed for specimens with and without interlayers, and the crack initiation, incremental crack propagation, and crack-stop concepts were introduced.

#### 2.2.1. FEA Model of Tensile Test and SENB Specimen

**Figure 3:**
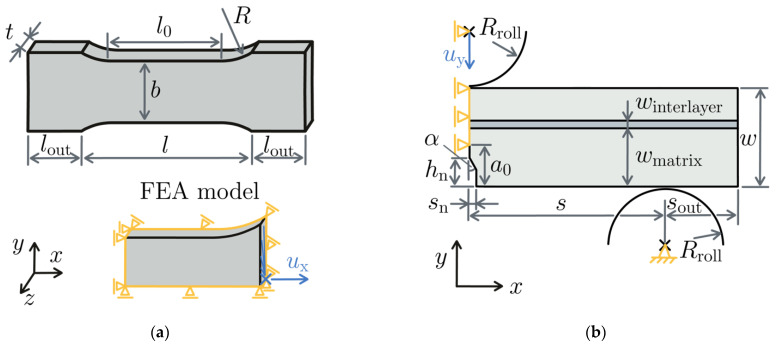
Model setup of (**a**) tensile model and (**b**) three-point bending model.

The geometry and model setup of the tensile test performed on HDPE specimens are defined in Figure 3a. The corresponding parameters are stated in Table 1. One-eighth of the geometry is modeled and the displacement $u_{x}$ is applied in the plane where the shoulders end. The model uses 20-node hexahedral elements with reduced integration. The mesh size was set to 0.5 mm and the implicit solver of Abaqus [29] was used. The output of the model is the force/displacement curve for stress–strain curves of the materials that were obtained in various regions of the specimen via DIC.

The model of the SENB tests is shown in Figure 3b and its parameters are defined in Table 1. One-half of the specimen is modeled in a 2d plane stress model. In the x = 0 plane, the x-displacements are constrained. The outer rollers and the central rollers are modeled rigidly. While the outer roller is fixed, the top roller is constrained in the x-direction, and a displacement $u_{y}$ is applied to it. The crack lies in the x-symmetry plane, so that cracks can be propagated by releasing the x-constraints in the region of crack propagation. The model uses 8-node plane stress elements with reduced integration. The mesh size is 0.2 mm and is refined by a factor of 5 towards the region of the crack. The total number of elements in the SENB test model is 5700. The implicit solver of Abaqus is used. Between the specimen and the rollers, hard contact with a penalty formulation is defined. The coefficient of friction μ between rollers and specimen was studied using the test results of the homogeneous SENB specimens.

The HDPE material was modeled using an elastic-plastic material model with isotropic hardening. The hardening curve was obtained from the tensile tests. No influence of the hydrostatic stress on the yield stress or the hardening behavior was accounted for. Since the EVOH material is considerably more brittle than HDPE, it was modeled linear-elastically in this work. The elastic parameters of both materials are listed in Table 1. For the crack initiation that is predicted in the EVOH layer, typical values for the critical stress and fracture toughness were chosen; see Table 1. Note that the critical stress in the combined criterion of Legullion [25] does not have to correspond to the ultimate tensile strength. The geometry and load parameters are indicated in Figure 3. *E*, *ν*, *σ*_c_, and *G*_c_ correspond to the Young’s modulus, Poisson’s ratio, critical stress, and critical energy release rate of the material, respectively. The SENB model uses the friction coefficient *µ* and the global mesh size *el*_size_, which is refined by the *factor* towards the crack tip.

#### 2.2.2. Incremental Crack Growth and Initiation Model

The model of the SENB specimen was set up to predict crack initiation and incremental crack propagation of the initial crack, in addition to the initiation of other cracks further ahead of the initial and previously initiated crack tips. The displacement was applied in displacement increments Δu. Each time the displacement was increased by Δu, the model was checked for crack initiation and propagation of the initial crack. Since plastic deformation occurs in vast regions of the specimen, common crack driving forces (CDF) from linear elastic fracture mechanics or even elastic-plastic fracture mechanics cannot be used. Therefore, an additional step, where the crack is extended by Δa, was computed and the change in the potential energy ΔU in the model was evaluated. This ΔU contains the whole strain energy, the plastic dissipation, and the frictional dissipation in the model, which are outputs of the FEA model. Since the displacement load $u_{y}$ is held constant during crack propagation, the work of the external forces is not needed to calculate the energy release rate of the crack as ΔU/(Δa t) with t as the thickness of the model. This method is computationally expensive because an additional FEA simulation is needed to obtain the CDF at each crack tip. Moreover, the direction of crack propagation must be known or assumed beforehand. Therefore, crack initiation and propagation, in addition to re-initiation, are regarded only in the x-plane of the SENB specimen.

If the CDF obtained in this way is above the fracture toughness $G_{c}$ of the material, the crack that was extended by Δa is regarded as the new, updated crack. If not, the computation with the extended crack is discarded.

In parallel, the model is checked for the initiation of additional cracks at different positions. In the layered SENB specimen, a crack can initiate in the x-plane from the bottom point of the EVOH layer upwards. This is due to the brittle behavior of the EVOH material compared to the HDPE material. For initiation, the combined stress-energy criterion as postulated in [25] is used, which states that a crack initiates when both a stress- and an energy criterion are fulfilled in a certain region. The stress can be directly evaluated in the FEA simulation and compared to a critical stress of the material. For the energy criterion, cracks with varied initiation length have to be introduced and computed. In the presented model, the stress criterion is checked first. If no crack can initiate based on the stress, the computationally costly energy criterion is not checked. The model starts with the longest possible crack where the stress criterion is fulfilled. Once a crack is initiated, it is regarded as an additional crack that can grow in an incremental manner. In this case, the SENB specimen has two crack tips that will subsequently be checked for crack growth.

The existing crack tips are repeatedly checked for crack propagation. Furthermore, initiation positions for new crack initiations are also monitored, until no crack tip propagates, and no initiation occurs. If this is the case, the displacement $u_{y}$ is increased by Δu and all crack tips and initiation positions are checked again.

#### 2.2.3. Advantages and Limitations of the Models

The proposed model uses the most general formulation for the crack driving force. Thus, it is very robust. Moreover, the principles of crack initiation and growth are based on physical principles and not empirical damage models. However, it is also very expensive with regard to computation times. In the presented case, only the use of a 2d model and a pre-known crack path allowed for reasonable computation times. Furthermore, the following simplifications and assumptions were made in the models:

- No delamination occurs between layers and the crack grows exactly in the symmetry plane of the SENB specimen.
- HDPE can be modeled elastic-plastically without any visco-plastic effects.
- The plastic deformation in EVOH can be neglected such that it can be modeled linear-elastically.
- The SENB test can be simplified as a 2d model with plane stress elements.
- The difference in the HDPE with and without carbon black can be neglected.

This shows that the model would need to be extended to be used for realistic components and more accurate results. For simplified layered geometries, toughening principles in heterogeneous material can be understood and predictions can be made with reasonable effort.

## 3. Results and Discussion

### 3.1. Material Model from Tensile Test

**Figure 4:**
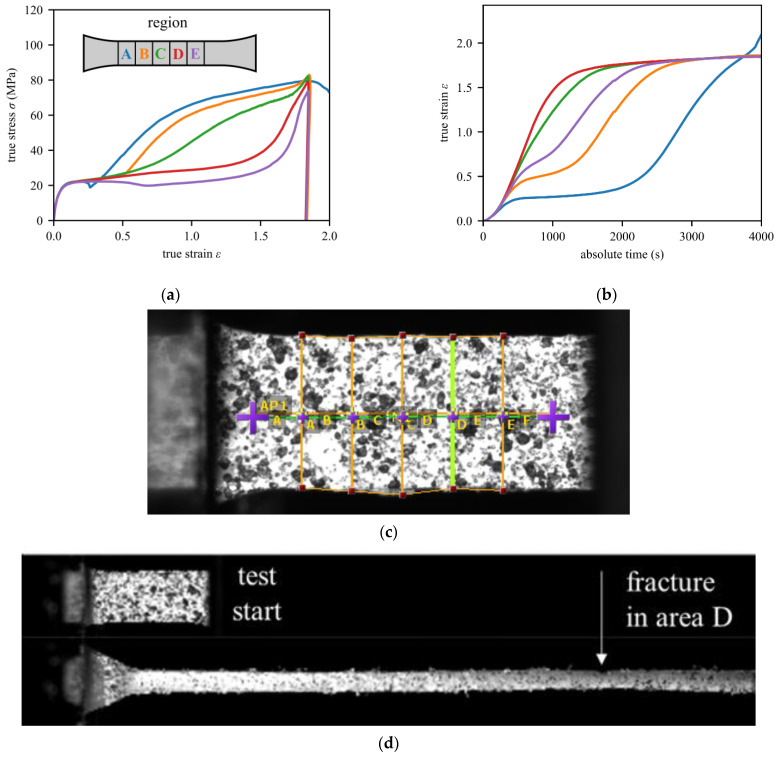
Results of the tensile test with a white specimen with true stress and strain computed using the DIC measurements. Plotted are (**a**) the stress–strain curve and (**b**) the strain–time curve to identify regions that reach high strains earlier. In (**c**), the actual speckle pattern and used evaluation regions are shown. Sub-figure (**d**) shows the front view of a tested sample at the test start and the final frame before failure, including the point of fracture onset in area D.

To fit a suitable elastic-plastic material model, accurate stress–strain curves of the HDPE material are needed. Digital image correlation (DIC) was used in the tensile tests with an analysis from the front of the specimen and the side of the specimen to compute the correct residual ligament area during testing, as shown in [30]. The five regions indicated in Figure 4a were defined, and the local strains were evaluated within these regions. By monitoring the change in the width and the thickness of the specimen in those regions, the average true stress can also be computed. Depending on the region of necking, significantly different stress–strain curves can be obtained for A to E. While areas within the area of necking (such as D and E) provide curve shapes that are typical for HDPE, areas on the outside (e.g., A and B) reflect significantly different curves and should not be used for further analysis. It can be assumed that the region with the earliest increase in the strains gives the most accurate stress–strain curves. In Figure 4b, such strain curves are plotted over time. This indicates that region C or D should be used. Figure 4c shows the regions on the actual sample, in addition to the used speckle pattern. In Figure 4d, the overall deformation behavior (first and last frame before failure) and the region of final fracture (region D) are shown.

For regions C and D, the hardening curves from Figure 4a were put into a multi-point elastic-plastic material model with isotropic hardening. For stress states different from a tensile test, true strains above 1.8 can be reached in the model. Thus, it is necessary to extrapolate the hardening curve from the experimental data to strains above 1.8 in the material model. The hardening curve is continued linearly to the point with a plastic strain of 500% and a stress of 500 MPa, which corresponds to the slope that the region C and D curves have at a strain of 1.8.

**Figure 5:**
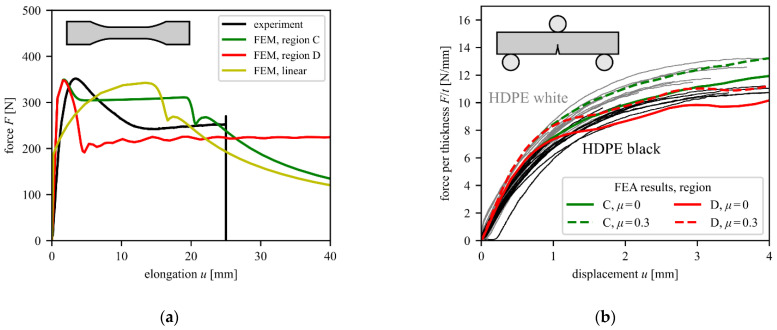
(**a**) Force–elongation curves of the tensile test and the corresponding FE simulation model using the fitted elastic-plastic material models and (**b**) study of the influence of the roller-specimen friction coefficient on the force–displacement curve of the SENB specimen with bulk white HDPE material. The continuous gray and black lines represent individual measurements to illustrate the expected scatter of experimental data.

To test the performance of the tensile curves obtained with the DIC measurements, the fitted material models were put into the model of the tensile test and the calculated load/displacement curves were compared to the experimental ones (see Figure 5a). For small elongations, the FEM results show a higher force than the experimental curves. The peak in the force agrees well between the tests and the FEM results with both plastic curves. After the start of necking, the force level of the model for region C is considerably higher than that for region D. Apart from the earlier drop in the force, the model for region D fits better with the tested curve and was thus used for further analysis. The discrepancies of FEM and test results indicate that the chosen elastic-plastic material model can only account for some of the effects that take place on the local scale of the material. A material model with a linear pressure-dependence of the yield stress (Drucker–Prager hardening model) was tested but did not show any improvement. Therefore, the difference may be due to viscous effects. Since the principal shape of the curve can be achieved with an elastic-plastic model, the huge effort of additionally obtaining time-dependent material properties was foregone.

To further evaluate the material models, including isotropic hardening and the data from regions C and D, the model was used for the single material SENB specimens. Figure 5b shows a comparison between the simulated and experimental data. The force is normalized to the thickness of each specimen. Furthermore, the coefficient of friction between the rollers and the specimen is varied. The results show that the experimental curves for the white HDPE specimens lay a little bit above the black HDPE specimens. The simulated curve that best approximates both white and black HDPE specimens uses an elastic-plastic material model based on region C and a friction coefficient of 0. The layered SENB specimens are thus simulated for the region C material model and without roller-specimen friction. The good agreement of experimental curves and the model results in Figure 5b indicates that the selection of the von Mises’ yield criterion and isotropic hardening is valid.

### 3.2. SENB Test and Model with Layered Material

**Figure 6:**
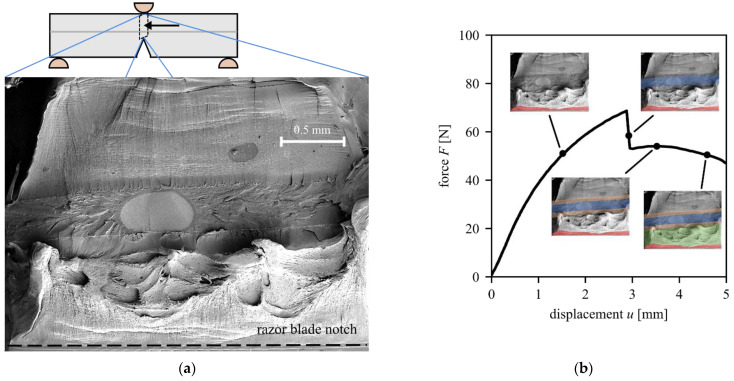
Experimental results of the SENB models with layered buildup: (**a**) fracture surface with distinct fracture regions and (**b**) force–displacement curve. The colored areas indicate the chronological order in which the fracture occurs on the specimen: red (crack tip blunting), blue (fracture of the EVOH layer), yellow (crack propagation from the EVOH layer), green (stretching and tearing of the remaining ligament between initial crack tip and fractured EVOH layer).

Figure 6 shows the results of a representative layered SENB specimen. Starting from the razor-blade notch at the bottom, the fracture surface is shown in Figure 6a. A distinct difference in the fracture surface can be noticed between the HDPE and the EVOH material. The HDPE material (bottom and top region of the fracture surface) features more plastic deformations and a more distinct tearing of the material. There seems to be an ellipsis-shaped starting defect in the EVOH layer in the center of the cross-section. Overall, EVOH shows a more brittle fracture behavior.

Figure 6b shows the force–displacement curve for this SENB specimen and associates points in this curve with the regions of the fracture surface (Figure 6a as miniaturized inlays) that have already cracked at that instant (colored regions). At the start of the test, the razor-blade notch represents the crack tip. With increasing load, this initial crack blunts and grows by about 0.1 mm. There is significant plastic deformation in the specimen, which is also indicated by the non-linearity of the *F*-*u* curve (see Figure 5b). At a force of approximately 65 N and a displacement of 3 mm, there is a drop of about 16 N in the force. This is due to an initiated crack in the brittle EVOH layer. From this point on, the initial crack at the bottom, and an additional crack that extends into the HDPE from the bottom and the top of the EVOH layer, exist. With increasing displacement, the force stays nearly constant, and this newly formed crack grows in both directions into the HDPE. The remaining ligament between the initial crack and the cracked EVOH layer deforms plastically when the displacement is further increased. To obtain the fracture surface in Figure 6a, the specimen was cooled using liquid nitrogen and broken as described in Section 2.1.3.

**Figure 7:**
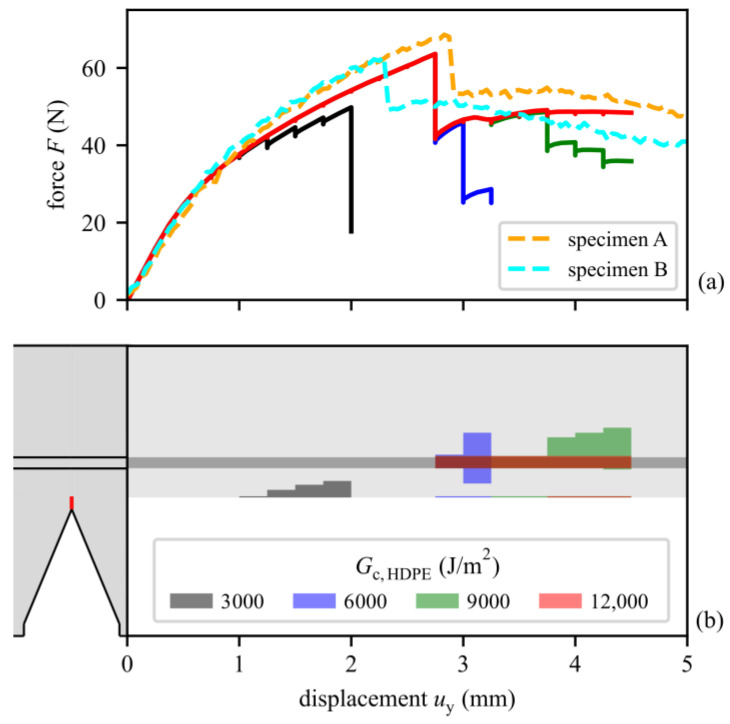
(**a**) Forces plotted over the displacement for the layered SENB specimens for both the numerical models and the experiments. For the simulation results, the critical energy release rate $\;G_{c,\mathrm{HDPE}}$ is varied between 3000 and 12,000 J/m^2^. Frictionless contact is used between roller and specimen. Additionally, (**b**) shows the corresponding cracked regions in the specimen as dark gray, blue, green, and red areas. For $\;G_{c,\mathrm{HDPE}}$ = 3000 J/m^2^, the initial crack grows into the HDPE material. For higher $\;G_{c,\mathrm{HDPE}}$ values, there is very small growth in the initial crack, and at a displacement *u*_y_ of 2.75 mm, a crack initiates in the EVOH layer and grows through the whole EVOH layer.

Figure 7a shows the experimental and the simulated force–displacement curves of the multi-layer SENB specimens. Results of two of the six tested specimens are shown. Furthermore, model results with a varied critical energy release rate *G*_c_, as a measure of the fracture toughness of the HDPE material between 3000 and 12,000 J/m^2^, are depicted.

Figure 7b plots the cracked regions over the applied displacement as dark gray, blue, green, or red regions. The EVOH interlayer is drawn as a dark gray region. For example, the force–displacement curve in Figure 7a for a $\;G_{c,\mathrm{HDPE}}$ value of 3000 J/m^2^ shows some kinks that start at a displacement of 1 mm. These kinks correspond to a growing crack in the HDPE material at the initial notch tip, as illustrated in Figure 7b as a dark gray area. For a displacement of 1.8 mm (left dashed blue line in the top diagram), a pictogram shows that the dark gray area corresponds to a crack from the bottom that is already approaching the EVOH layer. This is not representative of the real experiments, indicating that this value of $\;G_{c,\mathrm{HDPE}}$ is too low. For higher values of $G_{c,\mathrm{HDPE}}$, the first considerable cracks evolve in the EVOH layer and grow into the HDPE material from there. Since the fracture toughness and strength of the EVOH is not varied, the drop in the *F*-*u* curve is the same for $\;G_{c,\mathrm{HDPE}}\geq 6000\;J/m^{2}$. For a displacement of 4.25 mm and $\;G_{c,\mathrm{HDPE}}$ = 9000 J/m^2^, another pictogram shows how the initial crack has grown only to a small extent, but the crack that has initiated in the EVOH layer has grown further into the HDPE material, aligning with experimental observations. The overall shape of the *F*-*u* curves of the model and the experiments fit well for values of $\;G_{c,\mathrm{HDPE}}\geq 6000\;J/m^{2}$. The difference between the two experimental curves indicates the scattering in the experiment, which can be due to initial defects in the material, the variation in the geometry of the specimens, and the variation in the initial notch length after broaching. The shape of the experimental curves after the failure of the EVOH layer indicates that the suited critical energy release rate $G_{c,\mathrm{HDPE}}$ in the model is between 9000 and $12,000\;J/m^{2}$. For $G_{c,\mathrm{HDPE}}$ = 9000 J/m^2^, there is additional crack propagation for *u*_y_ > 3.75 mm (right dashed blue line), whereas for $G_{c,\mathrm{HDPE}}$ = 12,000 J/m^2^ there is no additional crack propagation. The decline in the experimental curves above *u*_y_ = 3 mm indicates that there is some more crack propagation in that region, but to a smaller extent than for $G_{c,\mathrm{HDPE}}$ = 9000 J/m^2^. In the experimental testing of the multilayer setup, a peak force for the failure of the EVOH layer of 61.4 ± 2.2 N was observed. The peak force observed in the simulation with optimized parameters ($G_{c,\mathrm{HDPE}}$ between 9000 and $12,000\;J/m^{2}$) was found to be 63.6 N. This indicates a difference between experiments and simulation of below 3.6%.

**Figure 8:**
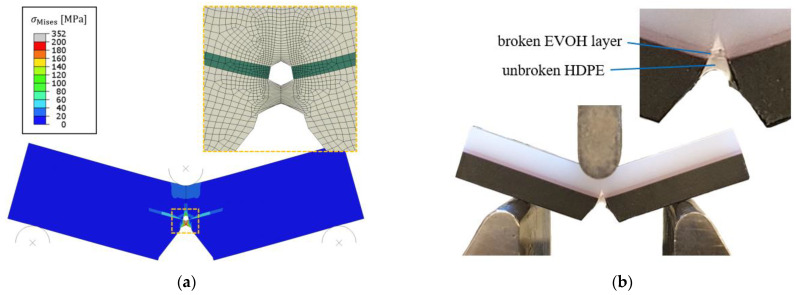
(**a**) Mises stress field (MPa) in the FEA model of the layered SENB specimen for a displacement load *u*_y_ of 4.5 mm and an HDPE fracture toughness of 12,000 J/m^2^, and (**b**) the specimen’s deformation and crack propagation in the experiment for *u*_y_ = 6 mm. The initial crack tip is blunted, and a crack has initiated in the EVOH layer that has slightly grown into the HDPE material.

In the description of Figure 6, a sequence of failing regions is stated that is based on the fracture surface. Figure 8 shows the model results (Figure 8a) and the experimental results (Figure 8b) of the SENB specimen for an applied displacement of 4.5 mm ($G_{c,\mathrm{HDPE}}$ = 12,000 J/m^2^) and 6 mm, respectively. Note that the displacement is lower in the simulation results because excessive deformations occurred in the elements for higher displacements. This displacement corresponds to the instance where the initial crack has blunted and the EVOH layer has fractured, which can clearly be seen in the model results. The same picture is visible for the experiment in Figure 8b. The HDPE ligament between the initial notch and the EVOH layer is highly stretched but still intact. In contrast, the EVOH layer is already fractured and the material of the HDPE layer behind the EVOH layer is already being plastically deformed and is blunting. This comparison shows that not only are the numerical results between the simulation and experiment comparable, but also that the overall fracture progression is identical.

## 4. Summary and Conclusions

The current work deals with the development of a simple methodology to predict both crack initiation and propagation in multi-layer materials. To do so, material parameters were determined with tensile test specimens using two cameras and DIC measurements. The acquired data were validated via re-computation of the performed tensile tests, and fracture mechanical tests on single material SENB specimens. After validation of the material parameter set, multi-layer SENB specimens that consist of two outer layers of HDPE and an interlayer of EVOH were tested. The results show that the force–displacement curve of the SENB specimen features a drop in force when a crack initiates in and propagates through the middle, more brittle EVOH layer. The remaining ligament between the initial crack of the SENB specimen and the brittle layer is plastically deformed and bridges the crack. This exact behavior was reproduced well with the finite element model that checks for crack initiation using the combined stress-energy criterion and for crack growth with an incremental energy approach. The experimental force–displacement curves were quite accurately reproduced using the 2d finite element model that used a von Mises’ yield criterion and no time-dependent material behavior. The difference in average peak force (that occurs when the EVOH layer fractures) between the experimental and numerical analysis was below 3.6%, showing the high potential of this methodology.

Combining the criterion for crack initiation and the energy concept for crack propagation in a 2d finite element model constitutes a useful tool for simulating the fracture progression in SENB specimens of layered materials that feature high plastic deformations. Since the modeling method is based on the fracture toughness and the critical stresses of the constituents only, this is a very general approach in terms of material behavior. However, this incremental crack growth model is limited to previously known crack paths and rather simple test setups. Moreover, extending the crack and performing a separate FE analysis each time the crack driving force is computed is not very efficient, even though it is valid for any extent of plastic deformation in the model. This means that the method can be used to point out interesting cases for simulation and provide starting points for developing more efficient modeling methods for crack initiation and growth, such as efficient configurational force-based crack propagation concepts (e.g., [31]) or meta-model-based crack-initiation methods [32]. Furthermore, future modeling of polymeric materials should also include the time dependency of the materials, in order to be more universally applicable.

## Figures and Tables

**Table 1:** Geometry, loading, and material parameters of the FEA models.

	Tensile Test								Materials					
**name**	*b*	*l*_0_	*T*	*R*	*l*	*l*_out_	*el*_size_	*u*_x_	*E*_HDPE_	*ν*_HDPE_	*E*_EVOH_	*ν*_EVOH_	*σ*_c,EVOH_	*G*_c,EVOH_
**value**	2.59	10	3.55	25	15	7	0.5	40	900	0.4	3000	0.3	140	1000
**unit**	mm	mm	Mm	mm	mm	mm	mm	mm	MPa	-	MPa	-	MPa	J/m^2^
**SENB test**														
*s*	*s*_out_	*w*	*T*	*R*_roll_	*w*_matrix_	*w*_interlayer_	*a*_0_	*h*_n_	*s_n_*	*α*	*u*_y_	*u*	*µ*_roll_	*el*_size_, *factor*
18	1.8	9.22	2.9	2	5.33	0.35	4.4	4	1.5	22.5	4.5	0.25	0.15	0.2, 5
mm	mm	mm	Mm	mm	mm	mm	mm	mm	mm	°	mm	mm	mm	-

## Data Availability

The authors agree to make data and materials supporting the results or analyses presented in their paper available upon reasonable request.
